# Mobile Web App Intervention to Promote Breast Cancer Screening Among American Indian Women in the Northern Plains: Feasibility and Efficacy Study

**DOI:** 10.2196/47851

**Published:** 2023-07-20

**Authors:** Soonhee Roh, Yeon-Shim Lee, DenYelle B Kenyon, Amy J Elliott, Daniel G Petereit, Anu Gaba, Hee Yun Lee

**Affiliations:** 1 Department of Social Work University of South Dakota Sioux Falls, SD United States; 2 School of Social Work San Francisco State University San Francisco, CA United States; 3 Sanford School of Medicine University of South Dakota Sioux Falls, SD United States; 4 Avera Research Institute, Avera Health Sioux Falls, SD United States; 5 Monument Health Cancer Care Institute Rapid City, SD United States; 6 Sanford Roger Maris Cancer Center University of North Dakota Fargo, ND United States; 7 School of Social Work University of Alabama Tuscaloosa, AL United States

**Keywords:** American Indian women, breast cancer, mammogram, mHealth, mobile web app intervention

## Abstract

**Background:**

Breast cancer is the most common cancer in the United States and the second leading cause of death for American Indian women. American Indian women have lower rates of breast cancer screening than other racial groups, and disparities in breast cancer mortality and survival rates persist among them. To address this critical need, a culturally appropriate, accessible, and personalized intervention is necessary to promote breast cancer screening among American Indian women. This study used mobile health principles to develop a mobile web app-based mammogram intervention (wMammogram) for American Indian women in a remote, rural community in the Northern Plains.

**Objective:**

This study aimed to assess the feasibility and efficacy of the wMammogram intervention, which was designed to motivate American Indian women to undergo breast cancer screening, as compared with the control group, who received an educational brochure.

**Methods:**

Using community-based participatory research (CBPR) principles and a multipronged recruitment strategy in a randomized controlled trial design, we developed the wMammogram intervention. This study involved 122 American Indian women aged between 40 and 70 years, who were randomly assigned to either the intervention group (n=62) or the control group (n=60). Those in the intervention group received personalized and culturally appropriate messages through a mobile web app, while those in the control group received an educational brochure. We measured outcomes such as mammogram receipt, intention to receive breast cancer screening after the intervention, and participants’ satisfaction with and acceptance of the intervention.

**Results:**

A significantly higher proportion of women who received the wMammogram intervention (26/62, 42%; *P*=.009) completed mammograms by the 6-month follow-up than the control group (12/60, 20%). The wMammogram intervention group, compared with the control group, reported significantly higher ratings on perceived effectiveness of the intervention (*t*_120_=−5.22; *P*<.001), increase in knowledge (*t*_120_=−4.75; *P*<.001), and satisfaction with the intervention (*t*_120_=−3.61; *P*<.001). Moreover, compared with the brochure group, the intervention group expressed greater intention to receive a mammogram in the future when it is due (62/62, 100% vs 51/60, 85%) and were more willing to recommend the intervention they received to their friends (61/62, 98.4% vs 54/60, 90%) with statistically significant differences.

**Conclusions:**

This study shows the feasibility and efficacy of the wMammogram intervention to promote breast cancer screening for American Indian women in a remote, rural community-based setting. Findings suggest that, with advancements in technology and the ubiquity of mobile devices, mobile web apps could serve as a valuable health intervention tool that builds upon low-cost technology and enhances accessibility and sustainability of preventive care to help reduce breast health disparities experienced in hard-to-reach American Indian populations.

**Trial Registration:**

ClinicalTrials.gov NCT05530603; https://clinicaltrials.gov/ct2/show/NCT05530603

## Introduction

### Overview

Globally, breast cancer is the most common cancer in women. Breast cancer accounts for about 30% of all new cancer cases in women each year in the United States [1]. Among American Indian women, it is the second-leading cause of mortality [2]. From 2013 to 2017, United States mortality rates declined among non-Hispanic White women, Hispanic, and African American women [3-6], whereas American Indian women have a 10% higher death rate when compared to non-Hispanic White women [2]. Although several health disparities afflict the estimated 7.2 million American Indians of the 574 tribal entities federally recognized in the United States, cancer is a critical issue for this underserved population [2,7]. Early detection of breast cancer through regular mammograms can reduce breast cancer mortality by up to 40% [3,8].

In the United States, cancer incidence rates for American Indian populations vary by geographic region [9,10] and considerable regional disparities in cancer incidences and mortality rates exist within this population [9,11,12]. In the 2018 National Health Interview Survey, a sample of White women reported 73.1% having at least 2-year interval mammography, compared to only 64.4% of American Indian women [13]. Death rates in the United States from breast cancer dropped 39% from 1989 to 2017; however, Northern Plains American Indian women (and American Indian women in general) did not experience the same decline [3,4,9-12,14-17]. This gap is primarily due to disproportionately low screening rates in the American Indian population [9,11,14-16,18].

The identified barriers to screening vary from cultural issues [19-24] (including medical mistrust [25] and perceived experiences of discrimination [26]), logistical issues (including low income [22,23], geographic isolation [11,12], lack of health insurance [23,24], transportation difficulties [3,23,27], lack of time [27], and limited health care accessibility [11,24,26]), knowledge issues (including inadequate knowledge about breast cancer and screening guidelines [15,24,27,28] and fear of mammography or possible results [24]), and general health risks (including obesity [19,24,27]). A significant number of women from the American Indian community reside in remote regions, which creates a hindrance for them to obtain mammography services due to the unavailability of such facilities in many Indian Health Service clinics and the distance they must travel. Compared to other ethnic groups, American Indian women must travel 2-3 times longer to reach breast imaging facilities [11,29,30], which might be a contributing factor to the disparities in breast cancer mortality rates. Additionally, the longer travel distances to receive treatment may also add to this discrepancy [29,31].

Despite the notable disparity in breast cancer rates, there has been minimal research aimed at intervening in this population. Thus, it is crucial to develop an intervention that is tailored to the culture, easily accessible, and personalized, which can help eliminate obstacles and encourage breast cancer screening among American Indian women. Some cancer prevention efforts geared toward American Indian women exist (ie, health educator-led workshops and education sessions, psychoeducational DVDs, talking circles, and printed material on screening guidelines) [12,22,23,29,32-35]; however, these interventions have had limited impact on improving breast cancer screening. Research shows that the conventional intervention of health message handouts is insufficient in producing the desired behavioral outcomes in cancer screening [22,23,32-35]. The key reasons for the limited screening behavioral outcomes include that American Indian women tend to be a hard-to-reach population [12,28,35-38] and past efforts have typically used a “one size fits all” approach rather than culturally tailored interventions that aim at the specific obstacles individual participants face [39-41].

There has been a call to develop customized intervention methods to decrease barriers and encourage screening while addressing the cultural differences among the 574 federally recognized tribes [7,9,15]. However, there has been limited success in creating interventions to address this issue. Capitalizing on the widespread use of web technologies, this pilot study developed a mobile web app-based educational intervention (wMammogram) to promote breast cancer screening among American Indian women. The wMammogram intervention delivers interactive and culturally tailored breast cancer care messages conveyed through the web and mobile devices to cover a wide range of topics and overcome known barriers for American Indian women.

### Conceptual Framework

The use of the internet in health interventions (mobile health [mHealth]) is on the rise, with research showing that it is an effective means of changing health behaviors. mHealth interventions have been applied to a wide range of health issues, such as weight loss, cancer screening, blood pressure, diabetes, mental wellness, physical activity, asthma, stroke, and smoking cessation [28,42-51]. However, previous mHealth interventions have been criticized for lacking scientific rigor [52], not being based on established theories [53], and failing to cater to individual needs [43]. To address these issues, the wMammogram intervention was developed using the Fogg Behavior Model (FBM) [54,55], which provides a framework for understanding human behavior in relation to technology. This intervention aims to call American Indian women to action to undergo mammograms through 3 stages: identifying barriers, developing motivators, and providing behavioral triggers. Specific barriers that prevent American Indian women from receiving mammograms are identified, and mobile tools, such as SMS or MMS (multimedia messaging service), are used to create customized motivators to improve knowledge. Finally, behavioral call-to-action reminders in the form of SMS text messages or electronic links are provided to prompt women to obtain mammograms.

### Objectives and Hypothesis

The purpose of this study was to examine the feasibility and efficacy of a culturally tailored mobile web app-based educational intervention (wMammogram) for American Indian women residing in a remote, rural area in the Northern Plains. To evaluate the feasibility, we assessed satisfaction with the wMammogram intervention 1 week after the intervention and measured efficacy by participants’ receipt (or not) of a mammogram 6 months after the intervention and their intent to undergo a mammogram 1 week after the intervention.

We hypothesized that participants in the intervention group would (1) report having received a mammogram at a higher rate, (2) demonstrate greater intention for receiving a mammogram in the future, (3) show more improved knowledge about cancer screening, and (4) express greater effectiveness and satisfaction with the intervention in comparison with participants in the control group. As no previous study has evaluated a mobile web app-based breast cancer screening intervention in this underserved population, this pilot study provides important insights into the feasibility and efficacy of the wMammogram intervention tool for creating awareness about breast cancer and screening.

## Methods

### Community Advisory Board

This study used a CBPR approach [56], which involved forming collaborative partnerships between the research team and the tribe. To achieve this, a community advisory board (CAB) was established, consisting of 8 community representatives and a multidisciplinary research team. The research team comprised American Indian health care professionals, behavioral scientists, and community leaders from faith-based organizations, academia, social service providers, and governmental organizations. The CAB members provided guidance to the research team in all aspects of the study, including the development, implementation, and dissemination of research findings. The CAB played a crucial role in generating content for the text, multimedia messages, and strategies for participant recruitment and retention. They also assisted in improving website accessibility and interpreting preliminary findings. Quarterly meetings were held by the CAB to obtain community involvement and insights regarding community concerns, assist in recruitment strategies, and ensure cultural relevance. CAB members were compensated US $50 for their time and received a gift card to cover their travel and participation expenses.

### wMammogram Intervention Development

After the CAB was formed, a series of focus groups were conducted with American Indian women aged between 40 and 70 years to identify barriers, motivators, and patterns of mobile phone usage [24]. The process and results of focus groups were detailed in a separate manuscript [24]. During these 2-hour sessions, American Indian participants in focus groups identified several barriers that are similar to those faced by non-American Indian populations when it comes to breast cancer screening. These barriers include cost, poverty, lack of health insurance coverage, fear of mammography or possible results, and privacy concerns. Additionally, participants living on reservations, where screening facilities do not exist, face inadequate access to screening facilities in their geographical area as well as a lack of transportation, which further inhibits their ability to undergo screening. Cultural attitudes also pose barriers to screening among American Indian participants. Some believe that only individuals with a family history of cancer are at risk for breast cancer or that the development of cancer is solely dependent on fate. Lack of understanding and awareness of the detailed procedure and recommended screening guidelines strongly influences their screening behaviors. Moreover, American Indian participants have a higher likelihood of mistrusting medical systems and experiencing discrimination, which makes them particularly vulnerable to not taking full advantage of mammography. Many of these barriers are modifiable, including health literacy, health access, and cultural attitudes and misconceptions. By targeting these modifiable barriers, the intervention aims to increase breast cancer screening rates among American Indian participants and reduce disparities in health care access and outcomes. Focus groups further identified the effective content, type, and frequency of messages to promote screening. Participants shared their current mobile phone habits and knowledge of breast cancer and screening guidelines.

The development of the mobile app, wMammogram, followed 4 steps: identifying barriers and mobile phone usage patterns, creating motivators, tailoring message web app content, and developing appropriate behavioral motivators and triggers. Data from the focus groups [24] and CAB were used to finalize the content and schedule of text messages, which were culturally tailored to address health beliefs and misconceptions about breast cancer screening. For example, culturally relevant imagery, music, and metaphors that emerged in focus groups were used to share Indigenous concepts of breast cancer and health. Visual messages, enhanced through the power of peers, featuring American Indian women sharing their personal experiences with breast cancer and screening were employed to challenge attitudes and beliefs about screening, such as the perception that breast cancer screening is unnecessary without symptoms, pains, or a family history of cancer. Scribed vignettes served as examples of screening test use, promoting participants’ self-efficacy in undergoing screening. Reminders were tailored to each participant’s preferred time and frequency.

The wMammogram system included 5 components designed to provide a personalized user experience: a web-based application for enrollment, setting user preferences, GPS navigation to nearby clinics, and message uploading; a database for participant records, rules, and messages; a program to determine message timing and content; an SMS text message delivery or reception platform; and a health navigator to offer cancer screening information, technical support, and transportation services. The user experience was interactive, with daily reminder messages adapted to each participant and varied throughout the week to keep messages fresh. Feedback from 3 usability tests of the wMammogram system prototype, conducted with 8 focus group participants before the randomized controlled trial, was incorporated into the final mobile app.

### Research Design

A randomized controlled trial (clinical trial registration ID: NCT05530603) was conducted over a period of 1 week with a 6-month follow-up to test the effectiveness of the wMammogram intervention. No blinding of participants or study personnel was implemented. The study involved 2 groups, with participants in the intervention group downloading the wMammogram mobile app on their personal mobile phones or using a phone lent by the research team. The control group received a printed brochure on breast cancer screening guidelines. Participants in both groups completed pre- and postintervention surveys with additional questions about the acceptability of the intervention. The surveys were conducted in person within 1 week of completing the intervention and by phone 6 months later. The study assessments were designed by the research team based on a literature review, input from CAB members, and the research team’s previous experience working with American Indian populations [15,24]. Trained and experienced American Indian female research assistants conducted all interview surveys.

### Participant Recruitment and Eligibility

**Figure 1:**
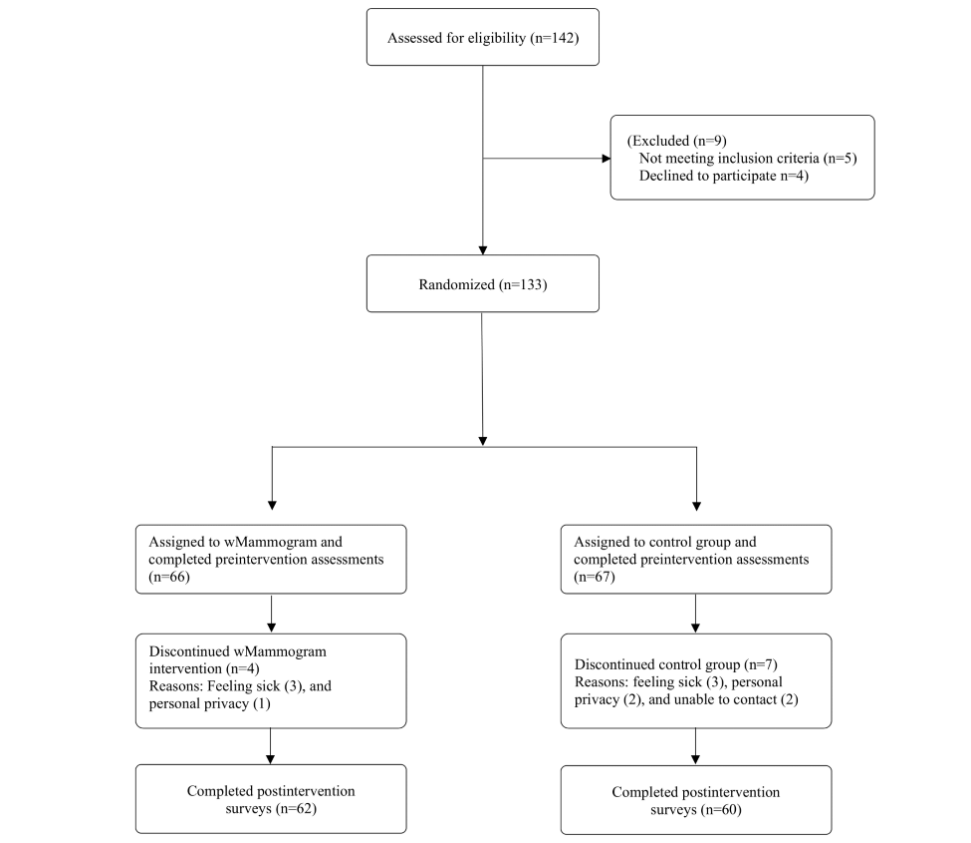
Flow diagram.

From October 2021 to December 2021, in a multipronged recruitment strategy, 142 American Indian women were recruited for participation, with 9 participants screened out of the study because of ineligibility (Figure 1).

We fully explained the purpose of this study, eligibility criteria, risks and benefits, confidentiality, and provided the contact information of the research team. Participants were also informed that their participation would be entirely voluntary and that they could withdraw at any time should they become uncomfortable with the study. Before the survey, all participants provided written, signed informed consent. After consenting, a total of 133 participants completed the pretest, while 122 participants completed both the pretest and posttest. The reasons participants did not complete the posttest include feeling sick during the intervention (n=6), unable to contact (n=2), and concern for their personal privacy (n=3). To be eligible for the study, participants were those who are self-identified American Indian women of the participating tribe in South Dakota, who are aged between 40 and 70 years, who have not received a mammogram in the past 2 years, and who are willing to use their own mobile phone, iPad, tablets, and computers, or a mobile phone borrowed from the research team for the wMammogram intervention. The age range for participants was selected based on the American Cancer Society breast cancer screening guidelines, which recommend women begin regular mammograms at the age of 45 years or at 40 years if they opt to start screening earlier [57]. Exclusion criteria include those who have received a mammogram in the past year and are younger than 40 or older than 70 years of age. Recruitment occurred through announcements (on tribal public radios, tribal public Facebook, and local newspapers), flyers (posted at community agencies, post offices, and religious organizations), referrals, and word of mouth. The announcement content specified the project purpose, eligibility criteria, and study personnel contact information. Interested individuals were screened by American Indian research assistants over the phone.

### Power Analysis, Attrition, and Randomization

To obtain an adequate sample size, we aimed to have 60 individuals in both the intervention and control groups at the 6-month follow-up assessment, considering a possible attrition rate of no more than 20% (n=24) [58]. The study initially had 142 participants, with 133 completing the pretest and 122 completing both the pre- and posttests. Randomization of group assignments was done in a 1:1 ratio for the 133 participants using sequentially numbered, opaque, sealed envelopes [59]. A total of 11 participants were excluded from data analysis due to failure to complete the posttest, resulting in an attrition rate of 8.27%. Specifically, in the intervention group, 4 participants discontinued taking part (resulting in a 6.06% attrition rate), whereas in the control group, 7 participants discontinued taking part (resulting in a 10.45% attrition rate). Each participant received US $10 in compensation for face-to-face interviews, with an additional US $20 for transportation. Those in the intervention group received US $10 each day during the weeklong intervention, US $10 for quiz rewards, and US $65 for mobile phone data fees over the 6-month period. All study participants received incentives ranging from US $40 to US $185 per person.

### wMammogram Intervention

Initially, face-to-face pretest surveys were conducted to gather information on breast cancer knowledge, barriers to screening, intentions to receive a mammogram, and personal preferences related to SMS and MMS. Participants’ personal risk of breast cancer was also assessed using a true or false questionnaire.

During the wMammogram intervention, participants received 2 to 3 messages per day for a week. Half of the messages required a reply to balance education and motivation. The messages aimed to increase knowledge and motivation around mammography and used various strategies to achieve this. Participants were incentivized to engage with the intervention by earning digital pink ribbons for each response to a question or prompt. Some messages were visual and included illustrations, photos, and videos featuring women sharing their experiences with mammogram screening.

To trigger behavior change, participants were asked questions at the end of topic-based message sequences. If a participant replied yes, they were sent links to clinics in their area and motivational statements to encourage them to make an appointment. A nurse health navigator was available to help participants access resources and transportation. After receiving the wMammogram intervention, participants were assessed twice: 1 week after the intervention participants filled out postsurveys and then were interviewed in 6-month follow-ups by the research team.

### Control Condition

Traditionally, to promote cancer screening, health service agencies mail printed material with the contact information of a health navigator for questions regarding the information provided in the brochure. The control group in this study received the same traditional approach, which included a brochure about breast cancer and screening guidelines from the American Cancer Society [57], as well as a list of community clinics that offer low-cost or free mammography. The assessment schedule for the control group was the same as for the intervention group, with testing taking place at baseline, 1 week after the intervention, and 6-month follow-ups, but without the actual intervention being given.

### Measures

#### Primary Outcome Measure

##### Mammography Receipt

The study’s primary outcome measure—whether a participant had received a mammogram—was evaluated at the 6-month follow-up by asking participants if they had undergone a mammogram after the wMammogram intervention. The assessment was based on self-reported answers of “*yes*” or “*no*,” which has been established as a reliable variable for evaluating the effectiveness of interventions in cancer screening research [28].

#### Secondary Outcome Measures

##### Intervention Effectiveness

A 4-point scale ranging from very ineffective (1) to very effective (4) was used to measure the intervention’s efficacy 1 week after the intervention.

##### Increase in Knowledge

Participants’ perceived level of knowledge about mammography was measured on a 3-point scale item (ranging from 1=same to 3=very improved) 1 week after the intervention.

##### Satisfaction With Intervention

Participant satisfaction regarding the intervention they received was assessed using a 4-point scale item ranging from very dissatisfied (1) to very satisfied (4) 1 week after the intervention.

##### Intention to Receive a Mammogram in the Future

Participants’ intention to receive a mammogram was measured using yes-or-no items 1 week after the intervention.

##### Recommendation of Mammography

Participants’ willingness to recommend receiving a mammogram to their friends was also measured using yes-or-no items one week after the intervention.

#### Background Variables

Based on existing research on behavioral health disparities [60], 3 sets of background variables were selected for this study.

##### Sociodemographic Characteristics

The variables of sociodemographic characteristics included age (continuous variable), education (ranged from 1=less than high school to 3=high school or general educational development [GED] or over), employment (1=yes; 0=no), income (ranged from 1=less than US $1499 to 3=US $3000 or more), birthplace (1=reservation; 0=nonreservation), and marital status (ranged from 0=single or never married to 2=divorced or separated).

##### Health- and Mental Health–Related Variables

Health status (ranged from 1=poor to 4=excellent) and mental health status (ranged from 1=poor to 4=excellent) were measured to assess participants’ health and mental health.

##### Lifestyle Variables

The variables of lifestyle characteristics included exercise (ranging from 0=not at all to 7=more than seven times of exercise per week), drinking (1=yes; 0=no), smoking (ranging from 0=not at all to 2=every day), and social activity (ranged from 0=none to 5=five or more per month).

### Data Analysis

The data analyzed included the 62 participants in the intervention (ie, wMammogram app) group and the 60 participants in the control group (ie, brochure) who completed pre and posttest questionnaires. The group equivalence in terms of baseline characteristics (ie, sociodemographics, physical health and mental health status, lifestyle variables related to exercise, drinking, smoking, and social activity) was examined using the *t* tests and chi-square tests. For hypothesis 1, the percentages of participants from each arm who received mammograms after the intervention were compared using the chi-square test. For hypotheses 2 through 4, the mean scores of intention to undergo a mammogram, improved knowledge, effectiveness, and satisfaction from each group were calculated and compared using the independent-samples *t* tests or chi-square tests. Furthermore, we estimated and compared the percentages of participants from each arm who responded “yes” to the recommendation of mammography using the chi-square test. Finally, a series of hierarchical logistic regression analyses were performed to determine the associations between mammography uptake and the wMammogram intervention while controlling for sociodemographic and health and mental health-related variables. All study data were anonymous and de-identified for confidentiality. All statistical analyses were conducted using SPSS 27 (IBM Corp).

### Ethics Approval

All study activities were approved by the tribal institutional review board and the institutional review board of the University of South Dakota (IRB- 2019-053).

## Results

### Sociodemographic and Baseline Measures of the Sample

Table 1 displays sociodemographic and baseline measures for continuous and categorical variables. The mean age of all participants was 52.89 (SD 10) years, and 59% (72/122) were born on the reservation. About 96.8% (118/122) had a high school education or GED or higher. Also, 49% (60/122) of the respondents made less than US $1499 monthly, and over half of the participants (67/122, 55%) were currently employed. Most participants (86/122, 71%) were single, never married, separated, divorced, or widowed. Regarding health status, about 49% (60/122) of respondents perceived that they had a “good” or “excellent” health condition, while 65.5% (80/122) reported being in a “good” or “excellent” mental health condition. On average, participants reported exercising at least 1.60 (SD 1.83) times a week. Almost 57% (70/122) were smokers, and 23% (28/122) were drinkers. The mean score of social activity was 0.96 (SD 1.41) per month.

Overall, the wMammogram app and brochure groups were significantly different in age (*P*=.003) and employment (*P*=.03). No significant differences were found on other variables, indicating that the 2 groups were initially similar in other sociodemographic and baseline measures.

**Table 1:** Baseline sociodemographic characteristics by group (N=122).

Variables		Web app intervention (n=62)	Brochure (n=60)	All (N=122)	Group difference					
					*t* test (*df*)		Chi-square (*df*)		*P* value	
Age (years), mean (SD)		50.50 (9.30)	55.37 (10.17)	52.89 (10.0)	2.76 (120.0)		N/A^a^		.003	
**Birthplace, n (%)**						N/A		0.79 (1.0)		.38
	Nonreservation	23 (37.1)	27 (45)	50 (41)						
	Reservation	39 (62.9)	33 (55)	72 (59)						
**Education, n (%)**						N/A		4.89 (2.0)		.09
	<High school	2 (3.2)	2 (3.3)	4 (3.3)						
	High school or general educational development	19 (30.6)	30 (50)	49 (40.2)						
	>High school or general educational development	41 (66.1)	28 (46.7)	69 (56.6)						
**Household income per month (US $), n (%)**						N/A		0.85 (2.0)		.66
	Less than 1499	28 (45.2)	32 (53.3)	60 (49.2)						
	1500-2999	19 (30.6)	15 (25)	34 (27.9)						
	3000 or more	15 (24.2)	13 (21.7)	28 (23)						
**Employment, n (%)**						N/A		4.69 (1.0)		.03
	No	22 (35.5)	33 (55)	55 (45.1)						
	Yes	40 (64.5)	27 (45)	67 (54.9)						
**Marital status, n (%)**						N/A		1.97 (2.0)		.37
	Single or never married	20 (32.8)	21 (35)	41 (33.9)						
	Married	21 (34.4)	14 (23.3)	35 (28.9)						
	Divorced or separated	20 (32.8)	25 (41.7)	45 (37.2)						
**Health status, n (%)**						N/A		6.63 (3.0)		.09
	Poor	1 (1.6)	8 (13.3)	9 (7.4)						
	Fair	29 (46.8)	24 (40)	53 (43.4)						
	Good	28 (45.2)	26 (43.3)	54 (44.3)						
	Excellent	4 (6.5)	2 (3.3)	6 (4.9)						
**Mental health status, n (%)**						N/A		4.32 (3.0)		.23
	Poor	3 (4.8)	7 (11.7)	10 (8.2)						
	Fair	18 (29)	14 (23.3)	32 (26.2)						
	Good	35 (56.5)	28 (46.7)	63 (51.6)						
	Excellent	6 (9.7)	11 (18.3)	17 (13.9)						
Number of exercise (times per week), mean (SD)		1.69 (1.81)	1.52 (1.86)	1.60 (1.83)	−0.52 (120.0)		N/A		.30	
**Smoking, n (%)**						N/A		0.27 (2.0)		.87
	Not at all	25 (40.3)	27 (45)	52 (42.6)						
	Some days	18 (29)	16 (26.7)	34 (27.9)						
	Everyday	19 (30.6)	17 (28.3)	36 (29.5)						
**Drinking, n (%)**						N/A		1.93 (1.0)		.16
	No	51 (82.3)	43 (71.7)	94 (77)						
	Yes	11 (17.7)	17 (28.3)	28 (23)						
Social activity (times per month), mean (SD)		1 (1.44)	0.92 (1.38)	0.96 (1.41)	−0.33 (120.0)		N/A		.75	

^a^N/A: not applicable.

### Hypothesis 1: Receipt of Mammography After the Intervention

The wMammogram group received mammograms significantly more than the brochure group after the intervention, as indicated in the chi-square test results (*χ*^2^_1_=6.8; *P*=.009) in Table 2. By the 6-month follow-up, 42% (26/62) of the wMammogram group participants versus 20% (12/60) of the brochure group participants had received a mammogram after the intervention. About 24% (15/62) of participants in the wMammogram app group and 12% (7/60) in the brochure group received a mammogram by themselves without any additional assistance. About 18% (11/62) of the wMammogram group participants and 8% (5/60) of the brochure group participants received a mammogram through local resource and information services (eg, All Women Count Program for those without health insurance or who were underinsured) arranged by the research team.

**Table 2:** Receipt of mammography after the intervention by group (N=122).

		Web app intervention (n=62), n (%)	Brochure (n=60), n (%)	All (N=122), n (%)	Group difference			
					Chi-square (*df*)		*P* value	
**Receipt of mammogram**						6.84 (1)		.009
	No	36 (58.1)	48 (80)	84 (68.9)				
	Yes	26 (41.9)	12 (20)	38 (31.1)				

### Hypotheses 2 Through 4: Effectiveness and Satisfaction of the Intervention

To test group differences in the effectiveness of and satisfaction with the intervention, we performed independent-sample *t* tests and chi-square tests for Likert-type items and dichotomous items, respectively (Table 3). As hypothesized, the wMammogram app intervention group demonstrated greater intention than the brochure group to receive a mammogram in the future when it is due (62/62, 100% vs 51/60, 85%), and these differences were statistically significant (hypothesis 2). Results also supported our hypotheses 3 and 4. Compared with the educational brochure group, the wMammogram app intervention group reported significantly higher levels of increased knowledge (*t*_120_=−4.75; *P*<.001), greater levels of perceived effectiveness of the intervention (*t*_120_=−5.22; *P*<.001), and greater levels of satisfaction with the intervention (*t*_120_=−3.61; *P*<.001). The wMammogram app intervention group further exhibited significantly greater willingness to recommend the intervention they received to their friends compared with the brochure group (61/62, 98.4% vs 54/60, 90%).

**Table 3:** Effectiveness and satisfaction of the intervention by group (N=122).

Variables		Web app intervention (n=62), n (%)	Brochure (n=60), n (%)	All (N=122), n (%)	Group difference					
					*t* test (*df*)		Chi-square (*df*)		*P* value	
**Effectiveness**						−5.22 (120.0)		N/A^a^		<.001
	Very ineffectual	0 (0)	4 (6.7)	4 (3.3)						
	Ineffectual	0 (0)	1 (1.7)	1 (0.8)						
	Effectual	9 (15)	30 (50)	39 (32)						
	Very effectual	53 (85)	25 (41.7)	78 (63.9)						
**Increase of knowledge**						−4.75 (120.0)		N/A		<.001
	Same	1 (1.6)	14 (23.3)	15 (12.3)						
	Improved	30 (48.4)	34 (56.7)	64 (52.5)						
	Very improved	31 (50)	12 (20)	43 (35.2)						
**Satisfaction with intervention**						−3.61 (120.0)		N/A		<.001
	Very dissatisfied	2 (3.2)	6 (10)	8 (6.6)						
	Dissatisfied	0 (0)	1 (1.7)	1 (0.8)						
	Satisfied	13 (21)	29 (48.3)	42 (34.4)						
	Very satisfied	47 (75.8)	24 (40)	71 (58.2)						
**Intention to receive a mammography in the future**						N/A		—^b^		.001
	Yes	62 (100)	51 (85)	113 (92.6)						
	No	0 (0)	9 (15)	9 (7.4)						
**Recommendation of mammography**						N/A		—^b^		.05
	Yes	61 (98.4)	54 (90)	115 (94.3)						
	No	1 (1.6)	6 (10)	7 (5.7)						

^a^N/A: not applicable.

^b^Not available. Instead of Pearson chi-square test, Fisher exact test was performed given that the expected count for some cells is less than 5. In SPSS, only the *P* value of the Fisher exact test is reported rather than the test statistic.

### Hierarchical Logistic Regressions

Table 4 presents the results of the hierarchical logistic regression models for undergoing a mammography screening test. In step 1, the intervention factor explained 6% of the variance in mammogram uptake. In step 2, participants’ sociodemographic characteristics accounted for 11% of the variance, which was an increase of 5% from step 1. In the final step, health- and mental health–related variables added an additional 4% to the explained variance, accounting for 15% of the variance in mammography uptake.

In step 1, the wMammogram intervention was a significant predictor of mammography uptake. American Indian women in the intervention group were significantly more likely to have received a mammography screening test (odds ratio [OR] 2.97; *P*≤.01). Step 2, where sociodemographic characteristics were added to step 1, had no statistical significance. In the final model (Step 3) of health- and mental health–related factors that were entered after controlling for the sociodemographic variables, the wMammogram intervention remained significant. American Indian women who participated in the wMammogram intervention group were 3.7 times more likely to have undergone a mammography test (OR 3.71; *P*≤.005) than those in the brochure group. The odds of receiving a mammogram screening test were greater for American Indian women who had better health status (OR 2.41; *P≤*.05).

**Table 4:** Hierarchical logistic regression model of receiving mammogram (N=122).

		Mammogram test step 1		Mammogram test step 2		Mammogram test step 3	
		Β^a^ (SE)	Exp (β)^b^	Β^a^ (SE)	Exp (β)^b^	Β^a^ (SE)	Exp (β)^b^
**Intervention**							
	wMammogram intervention	1.09 (0.41)**	2.97	1.36 (0.46)**	3.91	1.31 (0.47)**	3.71
**Socio** **economic status**		N/A^c^	N/A				
	Age			0.02 (0.02)	1.02	0.02 (0.02)	1.02
	Reservation-born			−0.54 (0.44)	0.58	−0.52 (0.46)	0.59
	Education			0.40 (1.22)	1.49	−0.45 (1.25)	0.96
	Employed			0.45 (0.52)	1.58	0.33 (0.55)	1.39
	Having health insurance			−0.09 (0.46)	0.91	−0.09 (0.48)	0.91
	Monthly income			−0.19 (0.13)	0.83	−0.24 (0.14)	0.79
	Married			−0.60 (0.50)	0.55	−0.50 (0.51)	0.61
**Health and mental health status**		N/A	N/A	N/A	N/A		
	Health status					0.88 (0.41)*	2.41
	Mental health status					−0.10 (0.36)	0.90
Model chi-square (*df*)		7.32 (10)**	N/A	14.31 (10)	N/A	20.19 (10)*	N/A
Cox and Snell *R*^2^		0.06	N/A	0.11	N/A	0.15	N/A

^a^Unstandardized Beta coefficients.

^b^Odds ratio.

^c^N/A: not applicable.

*P≤.05.

**P≤.01.

## Discussion

### Principal Findings

To the best of our knowledge, this research represents the first-ever endeavor to develop and assess a theory-based and CBPR-based wMammogram American Indian women’s education intervention. This study aimed to examine the feasibility and efficacy of the wMammogram intervention, which is culturally tailored for American Indian women residing in rural areas. The wMammogram intervention was successfully tested as per the trial protocols. The key finding was that the group that received the wMammogram intervention had a significantly higher rate of mammograms than the control group, indicating that web app interventions can positively influence health behaviors in the area of breast cancer screening. These results are consistent with previous mHealth projects [28,48,61,62] and add to the growing evidence that technology-based approaches can successfully address health concerns such as breast cancer screening, blood pressure control, diabetic self-management, physical activity promotion, stroke rehabilitation, weight loss, and other similar issues [28,44,46,48-51].

Due to a lack of culturally tailored interventions to promote cancer screenings for American Indian women, the first part of this study was focused on using CBPR methodologies to develop the wMammogram app in partnership with a Northern Plains tribe. This iterative process with the CAB and focus group participants [24] settled on a mobile web app intervention delivering 1 week of daily messages containing screening information (eg, guidelines) and cancer risk factors. The culturally tailored aspects of the messages, such as photos, videos, and messaging, were well received by participants.

The feasibility of the wMammogram intervention with this population of American Indian women appears to be strong, with 94% (62/66) of women in the intervention group completing the intervention. This is in line with what is seen in other cancer screening interventions [28,61,62]. Additionally, with data showing 100% of women logged in and completed 100% of quizzes with 85% correct response rates, it further bolsters confidence that American Indian women will engage in these types of intervention activities.

Satisfaction with the wMammogram intervention similarly appears to be overall positive, as 96.8% (60/62) of participants reported satisfaction with the intervention. More specifically, 98.4% (61/62) of participants had an increase in knowledge, and 85% (53/62) reported the intervention to be “very effective.” This pilot study did not evaluate how much (if any) of participants’ satisfaction with the intervention was related to the cultural tailoring of the message. Future studies should evaluate the degree to which culturally tailored messages contribute to intervention efficacy.

The efficacy of the wMammogram intervention appears to be strong, with the most exciting part being the documented effectiveness of the intervention on actual screening practices. Within the 6-month postintervention window, 42% (26/62) of the wMammogram app group participants obtained a mammogram. This statistically significant difference between the wMammogram group (26/62, 42%) and the control group (12/60, 20%) is commendable as a convenient and minimally time-intensive intervention. Additionally, further solidifying the perceived effectiveness of the wMammogram intervention, 100% of the participants expressed greater intention to receive a mammogram in the future, and 98.4% (61/62) were willing to recommend the intervention they received to their friends compared with the brochure group. These differences were statistically significant, and these rates are comparable to or exceed the rates of other technology-based cancer screening promotion interventions [28,61]. Furthermore, the wMammogram intervention increased the likelihood of receipt of mammogram screening by 3.7 times in this study. This research successfully demonstrated that it is possible to effectively recruit American Indian women for mHealth intervention research while also showing that the intervention was feasible and effective. This study contributed to the growing body of research that suggests American Indian women tend to use mobile technologies frequently and are open to mHealth interventions.

Ultimately, this research can serve as a framework for developing SMS and MMS message programs that prioritize the voices of the targeted population. Previous studies [63,64] that developed and revised messages in collaboration with health professionals and consumers may have missed important topics or experiences that are significant to research participants, which could limit the impact of the program and its delivery. In contrast, this study engaged participants, CAB members, researchers, and health professionals simultaneously throughout the process of creating, reviewing, and refining messages. All participants had an equal opportunity to offer their insights on the same initial messages, and the researchers focused on the ratings provided by usability test participants and CAB members when refining the content of the messages. Working with CABs can enhance user engagement and acceptance, as demonstrated in previous research [24,28], which could explain why research participants rated the message’s usefulness, feasibility, and efficacy so highly. These results have the potential to benefit other racial, ethnic, and American Indian tribal communities that aim to reduce health disparities by promoting adherence to screening guidelines.

### Strengths and Limitations

Due to the absence of research on breast cancer screening using mHealth interventions among American Indian women, this study has strengths as well as limitations. To the researcher’s knowledge, this is the first study to examine whether breast cancer screening can be improved using an mHealth intervention among rural American Indian women. This project was innovative in using the novel mHealth approach to develop a theory-driven intervention model based on the FBM [54,55] to improve breast cancer screening and lay the foundation for a cost-effective and accessible intervention, especially for isolated, hard-to-reach populations. Unlike a mobile phone intervention, the wMammogram intervention can be used by smartphone users and users of other devices such as computers, iPads, tablets, or Androids. According to a report issued by the National Indigenous Elder Justice Initiative [65] as well as the Fort Randall Telephone Company (Company Representative, email communication, August 16, 2019), most American Indian women residing in rural areas report using at least a computer, iPad, tablet, Android, or smartphone, which increases the reach of the wMammogram intervention to those with low income or less up-to-date technologies, enabling them to capitalize on their readily available resources. Because the wMammogram intervention increased breast cancer screening behavior and American Indian women’s adherence to mammogram guidelines, the developed intervention model enables the creation of tailored campaigns with wide applicability for replication to other underserved populations. The significance of this project is that it shifts the focus from the problem (breast cancer screening disparities) to identifying an evidence-based best practice that improves breast cancer screening among American Indian women. Many studies on breast cancer screening among American Indian women, rather than seeking solutions, focus on problems and primarily document the lower rates of breast cancer screening among American Indian women. Studies rarely translate, implement, and investigate an intervention’s efficacy, which this project does. Additionally, this project notably explores the complex factors that impact breast cancer screening among American Indian women. The breast cancer screening experience for American Indian women is greatly influenced by a range of factors including personal, cultural, and societal contexts such as poverty, limited access to health care due to geographic isolation, and differing cultural attitudes toward screening. The insights gleaned from this study are invaluable for practical and scholarly translational studies as well as for designing an intervention model that effectively addresses multiple barriers and diverse determinants.

Although the study was successful in motivating behavioral change, there are some limitations that need to be considered. First, the data collection method used a self-report survey, which means that participants may have provided answers that they believed were socially desirable rather than reflective of their actual thoughts or knowledge. Second, there were potential confounding factors that may have affected mammogram receipt in both groups due to cultural norms and the rapport between the research team and participants. The monthly phone calls to check whether participants had received a mammogram could have influenced participants’ behavior based on their relationship with the research team, and there is also a cultural norm for American Indian women to avoid giving a direct negative response. Third, providing health navigator services to the intervention and control groups may have contributed to the effect of the intervention on primary and secondary outcomes, and it is important to note the role of health navigator services in promoting mammography for American Indian women. Hence, future studies should use a 3-arm design to tease out the efficacy of the mobile web app intervention compared to the intervention with health navigation services and usual care. Fourth, the findings may have been affected by selection bias, as women who did not choose to participate in the study may have been less willing to discuss breast cancer screening than those who did. Additionally, the study did not include women preparing for surgery, and common protective factors (eg, social support, spirituality, and resilience) were not considered. Future studies with more representative samples of American Indian women could provide a fuller understanding of breast cancer screening effects, and such studies should also consider diverse tribal affiliations, regional location, and rural and urban contexts. Fifth, although 100% of intervention group participants expressed their intention to receive breast cancer screening, the measurement is based on the potential intention. In a real setting, the number could be going down in a larger population. Sixth, while CBPR with a specific Indigenous community may limit generalizability, the findings provide vital insights into the efficacy and feasibility of the mobile web app intervention for women within that tribe. This adaptable approach can be readily implemented for other Indigenous subgroups, leveraging the widespread accessibility and affordability of mobile web app services. Finally, our reliance on binary measurements of health behaviors has limited our insights and value. Future research must use more fine-grained measurements to unlock a wealth of knowledge and understand the nuanced timing and effectiveness of interventions.

### Conclusions

This study successfully shows the feasibility of a CBPR approach to recruiting American Indian women into mHealth intervention research. This study also contributes to the growing body of evidence that American Indian women are high users of mobile technologies and suggests they are likely to be receptive to mHealth interventions. The overall positive results give confidence in using cell phone technology to deliver important health promotion messages, particularly for recommended health screenings. Public health efforts with American Indian women should be sensitive to possible technology barriers, particularly in rural and reservation communities, but this study adds to the growing literature that adults aged 40-70 years will use and appreciate health information delivered through a mobile app.

Moving forward, it is essential for future research to explore how these methods can be further tailored to cater to different groups, with the aim of mobilizing women as health promoters and encouraging their loved ones to undergo screenings. To achieve equitable and accessible health care for all, it is imperative that future studies prioritize and consistently implement interventions guided by CBPR principles. This approach will enable us to effectively address the specific challenges faced by communities in accessing preventive health care services and pave the way for improved health outcomes.

## Abbreviations

CAB: community advisory board
CBPR: community-based participatory research
FBM: Fogg Behavior Model
GED: general educational development
mHealth: mobile health
MMS: multimedia messaging service
wMammogram: mobile web app-based mammogram

## Data Availability

The data sets analyzed during this study are not available.
